# Ending of the COVID-19 Related Public and National Health Emergency Declarations: Implications for Medically Underserved Populations in Tennessee

**DOI:** 10.26502/aimr.0164

**Published:** 2024-03-01

**Authors:** Donald J. Alcendor, Patricia Matthews-Juarez, Duane Smoot, James E.K. Hildreth, Paul D. Juarez

**Affiliations:** 1 Department of Microbiology, Immunology and Physiology, Center for AIDS Health Disparities Research, School of Medicine, Meharry Medical College, 1005 Dr. D.B. Todd Jr. Blvd., Nashville, TN, 37208-3599, USA; 2 Center for AIDS Health Disparities Research, Department of Microbiology, Immunology, and Physiology, School of Medicine, Meharry Medical College, 1005 Dr. D.B. Todd Jr. Blvd., Nashville, TN, USA; 3 Department of Family & Community Medicine, Meharry Medical College, 1005 D.B. Todd Jr. Blvd., Nashville, TN 37208, USA; 4 Department of Internal Medicine, School of Medicine, Meharry Medical College, 1005 D.B. Todd Jr. Blvd., Nashville, TN 37208, USA

**Keywords:** COVID-19, Tennessee, Emergency declarations, Uninsured, medically underserved, disparities

## Abstract

The Biden administration decided to end the COVID-19 National and Public Health emergencies on May 11, 2023. These emergency declarations were established by the Trump Administration in early 2020. Under the COVID-19 emergency declarations, US citizens were provided with COVID-19 testing, vaccines, and treatments at little or no cost. The declarations allowed the federal government the option of waiving and or modifying government programs such Medicare, Medicaid. The emergency declarations were directly tied to other COVID-19 related provisions that have also expired that includes Economic Security (CARES) Act, the American Rescue Plan Act (ARPA), the Families First Coronavirus Response Act (FFCRA), the Coronavirus Aid, Relief, and the Inflation Reduction Act (IRA), the Consolidated Appropriations Act, 2023 (CAA). In addition, there were other federal and state emergency programs that were provided and too numerous to report here. At the time of this writing, the state of Tennessee continues to have moderate and sporadic spikes in COVID-19 cases and hospitalizations. Tennessee has higher than the national average of uninsured and underinsured people in the US. In Tennessee, more than 600,000 people are uninsured or underinsured in 2023 according to a study by the Kaiser Family Foundation. The ending of the PHE greatly impact coverage, cost, and access to COVID related services that will disproportionately affect the uninsured and medically underserved populations in Tennessee, the south in general, and throughout the US. Medically underserved populations are those groups with disparities in primary care, living in poverty, older, or having higher than expected infant mortality.

## 1. Introduction.

The emergency declarations were in direct response to World Health Organization declaration of the COVID-19 pandemic on May 11, 2020 [1–4]. The Public and National emergency declarations during the pandemic have altered the impact of the pandemic since the peak of January 2022 [2]. As of February 9, 2023, approximately 270 million US citizens have received at least one dose of a COVID-19 vaccine. There has been a decline in COVID-19 cases of 92% and a subsequent decline in COVID-19 associated mortality of greater than over 80% [2]. The emergency declarations have been instrumental in providing COVID-19 treatments, vaccines, and related services. Now that the declarations have expired, the financial burden will disproportionately impact financially distressed populations in Tennessee where 10.1% of the population was uninsured in 2021 [5]. Tennessee ranks 9th in the US for uninsured persons [6]. Studies show that more than Approximately 800,000 Tennesseans (11%) are without insurance coverage [7]. In the nation, Tennessee ranked 4th among southern states for age adjusted death rates due to COVID-19 per 100,000 in the 3rd quarter of 2022 [8]. Tennessee ranks 8th in the US in overall deaths due to COVID-19, [9]. Among Southern states Tennessee consistently ranks worst in the US in overall health along with high rates of premature death and unmanaged chronic diseases [10–12]. High rates of smoking and sedentary lifestyles have contributed to high rates of obesity in southern states [13]. Southern states, especially in rural counties have less access to primary providers [14]. The disparities in health care in Tennessee have been exacerbated by the COVID pandemic. Unmanaged chronic diseases are more prevalent in the South and risk factors for severe COVID-19 disease. These uninsured communities in Tennessee that are least able to afford COVID services will be disproportionately burdened and may be forced to forgo medical care due to cost resulting in increased exposure, community spread, hospitalizations, and death especially among the most vulnerable individuals with chronic underlying clinical conditions.

### Research Problem

The ending of the COVID-19 related Public and National Health emergency declarations will greatly impact under-78 served populations in Tennessee.

### Ending the National and Public Health Emergencies needs to be addressed now

The ending of the emergency declarations needs to be addressed now because the COVID-19 pandemic is ongoing and 82 continues to disproportionately affect underserved populations who have no medical insurance, live in poverty, and 83 are least able to afford COVID-19 vaccines and related services. Maintaining some form of these declarations during 84 this different phase of the pandemic is essential for the health and wellness of these populations. This work will help 85 to define the PHE and NHE declarations, what these declarations accomplished, their significance to the development of the COVID-19 vaccines and funding for COVID related services, as well as their significance to public health especially for populations experiencing poverty and are uninsured in Tennessee.

## 2. Difference between a Public Health Emergency and a National Health Emergency

**Figure 1:**
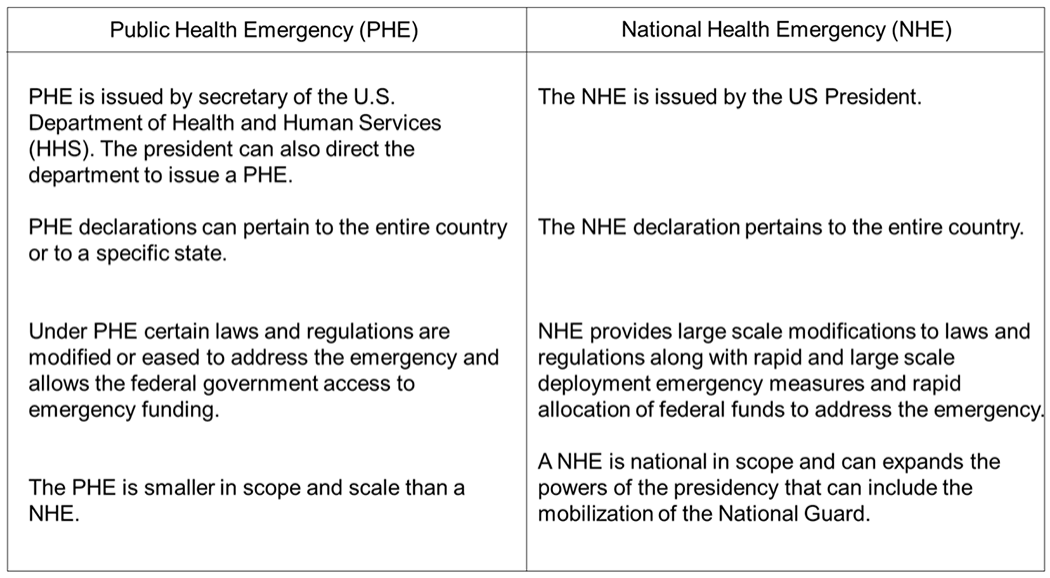
Major difference between the public and National health emergency declarations

There are three ways in which the federal government can declare emergency declarations, via the secretary of HHS, the President of the US, and a disaster declared under the Stafford Act. [15]. These declarations provide for different types of responses and can be mobilized consecutively. PHE emergency declarations are issued directly from the HHS secretary or recommended by the President to HHS. The PHE declaration can apply to the entire US or to a specific state. During a PHE federal and state guidelines are waived or modified to allow access to financial resources from the government that are needed to meet public and private needs during the time of the emergency [15]. A PHE is smaller in scope when compared to a national health emergency. During a major disaster or public health emergencies the federal government has the option to enact the Stafford Act that affects both state and tribal communities. Under the Stafford Act the governor or chief executive may request federal assistance for emergency and disaster relief [16]. Under the provisions of the Stafford Act the federal government can provide financial assistance to communities experiencing public health emergencies and disasters declared by the president of the US [15]. Additional provisions of the Stafford Act allow the Federal Emergency Management Agency (FEMA) to coordinate disaster relief to affected states. A National Health Emergency (NHE) can mobilize large-scale financial resources from the government in a timely manner. The president has extended powers during an NHE such as mobilizing the National Guard to support public health and safety [15]. Major differences between the COVID-19 Public and National health emergency declarations are shown in Figure 1.

## 3. Major Emergency declarations enacted during the COVID-19 pandemic

COVID-19 Emergency declarations that were declared in response to the COVID-19 pandemic, the federal government made emergency declarations under each of the following emergency authorities. On January 31, 2020, the Secretary declared a PHE regarding COVID-19 under Section 319 of the PHSA. This allowed state, tribal, and local health departments greater flexibility to request HHS to authorize them to temporarily assign personnel to respond to the COVID-19 outbreak if their salaries are normally funded in whole or in part by the PHSA programs [17]. Once again, on March 13, 2020, the President declared a COVID-related emergency under Section 501(b) of the Stafford Act [18]. Under the Act FEMA supplement efforts of HHS, including the Centers for Medicare & Medicaid Services (CMS) and the Centers for Disease Control and Prevention (White House 2020). This includes emergency protective measures taken in response to the COVID-19 outbreak. Under this provision, FEMA assistance is provided to states with a 75% federal match for disaster-related costs such as cost of state emergency operation centers, use of the National Guard, law enforcement, and other measures necessary to protect public health and safety [18].

**Figure 2:**
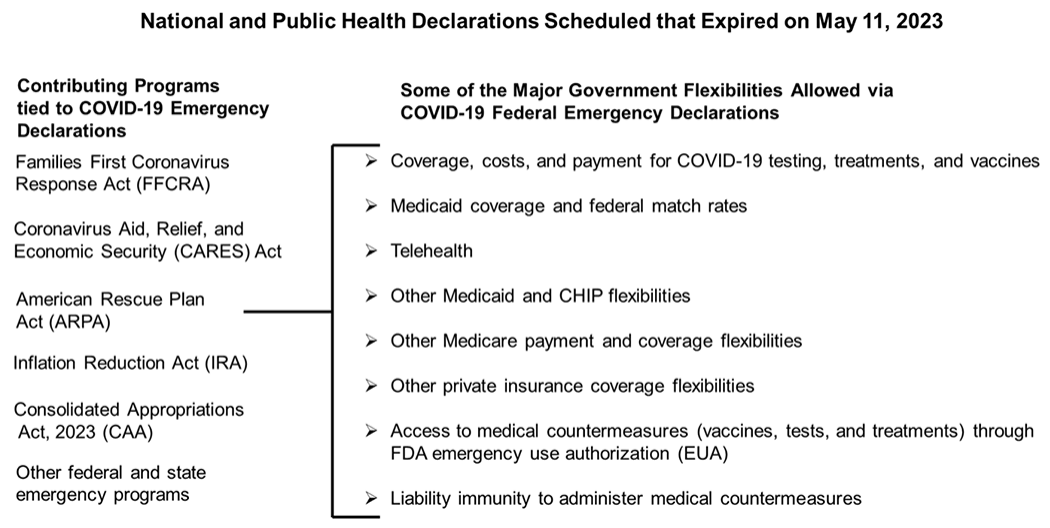
Major government flexibilities allowed during the COVID Public and National Health emergency declarations and the contributing programs.

On March 13, 2020, the President declared a national emergency beginning March 1 due to the COVID-19 outbreak under sections 201 and 301 of the National Emergencies Act and Section 1135 of the Social Security Act. The declaration directs the Secretary to exercise authority under Section 1135 of the Act to temporarily waive or modify certain requirements related to Medicare, Medicaid, and State Children’s Health Insurance Program. The Secretary may also waive authorities related to the Health Insurance Portability and Accountability Act (P.L. 104-191) [19]. Some of the major government flexibilities allowed during the COVID-19 federal health emergencies and their contributing programs are shown in Figure 2.

## 4. COVID-19 Public and National Emergency Declarations

### The COVID-19 Public Health Emergency Declaration (PHE)

The first US case of SARS-CoV-2 (COVID-19) infection was confirmed on January 20, 2020 and on January 27, 2020, the Secretary of Health and Human Services officially declared a public health emergency and activated new authorities for regulators and financial resources to support public health to contain this novel pathogen [20,21]. The PHSA secretary under Section can declare a Public Health Emergency after consultations with health officials involving a disorder or disease that presents a public health emergency including significant outbreaks of an infectious disease or a bioterrorist attack. The declaration does not require a formal request from the state or local government. Once PHE is declared the US Secretary can conduct and support investigations into the causes, treatments, and support prevention of the disease or disorders and enhance public health policies that can include issuing grants, modifying the practice of telemedicine, enter into state and government contracts, make temporary personnel available to respond to the emergency, grant an extension or waiver requirements of certain substance abuse and mental health services administration grants on a state-by-state basis, have access to the public health emergency fund, and grant extensions or waive sanctions related to submission of data or reports required by statute. HHS Secretary Alex Azar declared the recently expired PHE declaration on January 31, 2020 U [22]. Under Secretary Azar, the PREP Act emergency declaration was also enacted. The PREP Act provided liability protections and extensive preemption of conflicting state-based laws, to aid in facilitation of real-time manufacturing of qualified new medical countermeasures through public private partner-ships. This PHE was enacted via the Public Health Service Act under Section 319 and must be renewed every 3 months. The PHE has been renewed many times by both administrations but expired on May 11, 2023. The (PHE) for COVID-19 declared under section 319 of the Public Health Service Act, is not the same as the COVID-19 National Emergency declared by the Trump Administration in 2020 and implicated by H.J.Res.7 [23]. The current functions of HHS for COVID-19 would not be constrained by the ending of the National emergency declarations [23]. Existing mandates in effects under the authorization 1135 would remain in effect until the end of the PHE for COVID-19 [23].

### The COVID-19 National Health Emergency

A national emergency was issued by the Trump administration in March of 2020 under 168 the Stafford Act [24]. The Biden administration extended these declarations that 169 broaden the federal response to the pandemic under DHS and DoD and other government agencies [24] The national emergency declaration declared under the Trump administration was, National Emergencies Act pursuant to Section 201. This declaration can only be terminated by the current President Biden or by an act of Congress. The President can also renew the declarations annually.

## 5. Conditions in the US that warranted emergency action for COVID-19

On December 2019, a novel human beta-coronavirus known as severe acute respiratory syndrome coronavirus 2 (SARS-CoV-2), the virus causes COVID-19, was first detected in Wuhan, Hubei Province in the People’s Republic of China in a cluster of patients with pneumonia of unknown cause [25–27]. This new virus was observed to be asymptomatic in healthy people without underlying comorbidities but caused severe life threatening and multi systemic disease in individuals that were immunosuppressed or had underlying clinical disease [28,29]. This new human coronavirus was found to be highly contagious and could spread mainly via respiratory secretions and was highly transmissible upon close contact. In addition, infections resulted in increased hospitalizations and death especially among the elderly and immunocompromised populations [30]. There was no treatment of vaccines in the early days of the pandemic and because the virus was thought to evolve from bats, there was the potential for genetic variants that produce more severe disease. On January 31, 2020, a PHE declaration under section 319 of the Public Health Service Act (42 U.S.C. 247d), in response to COVID-19. In the US the PHE provisions included suspending entry of foreign nationals seeking entry who had been physically present within the prior 14 days in certain jurisdictions where COVID-19 outbreaks have occurred, including the People’s Republic of China, the Islamic Republic of Iran, and the Schengen Area of Europe [31]. Federal, State and local governments carried out emergency measures to slow the spread of the virus and treat those affected. This included instituting federal quarantines for individuals evacuated from foreign nations, issuing a declaration pursuant to section 319F-3 of the Public Health Service Act (42 U.S.C. 247d-6d), and releasing policies to accelerate the acquisition of personal protective equipment and streamline bringing new diagnostic capabilities to laboratories. On March 11, 2020, the World Health Organization (WHO) announced that the COVID-19 outbreak was a pandemic that resulted in an exponential increase in new infections in the US and around the world [32]. The spread of COVID-19 within our Nation’s communities threatens to strain our Nation’s healthcare systems. As of March 12, 2020, 1,645 people from 47 States have been infected with the virus that causes COVID-19. It is incumbent on hospitals and medical facilities throughout the country to assess their preparedness posture and be prepared to surge capacity and capability. Additional measures, however, are needed to successfully contain and combat the virus in the United States. The national emergency was declared on March 13, 2020 as part of an aggressive response to the COVID-19 pandemic. At the time, the Trump administration put in place aggressive regulatory flexibilities to curtail the spread of the novel coronavirus SARS-CoV-2 the established etiological agent for COVID-19 [33]. The emergency declaration gave medical providers, healthcare facilities, and states maximum flexibility to support public health in the US. Under the declaration, the Secretary of Health and Human Services (HHS) authorized the Centers for Medicare & Medicaid (CMS) to establish waivers to expand COVID-19 treatment and prevention services under the guidance of the White House Coronavirus Task Force. Under Section 1135 of the Social Security Act the president’s declaration allowed the HHS Secretary to waive certain Medicare, Medicaid and Children’s Health Insurance Program (CHIP) program as well as other safety net programs [34].

## 6. Conditions in the US that warrants ending of PHE and NHE for COVID-19

In the US over the past three years, there has been a historic sustained response to the COVID-19 pandemic. There has been more than 5 trillion dollars spent to support individuals and families, businesses, state and local governments including schools, and the healthcare industry [35]. We have more tools and resources than ever before including new ways to manage hospitalized patients, widespread availability of the new Omicron Bivalent booster, the antiviral Paxolvid, and we have at least 270 million people in the US have been immunized with one dose of a COVID-19 vaccine [36,37]. As of April 26, 2023, the Centers for Disease Control (CDC) reported approximately 104 million cases of COVID along with more than 6 million hospitalizations and 1.1 million deaths [38]. In 2020 and 2021, COVID-19 was reported by the CDC to be the third leading cause of death. In addition, COVID-19 was found to be the fourth leading cause of death in 2022 by the CDC [39]. During the pandemic at the time of this writing more than 675 million COVID-19 vaccinations have been given including more 55 million update Omicron bivalent boosters. In the US, we have reached an estimated 95% population immunity as of December 2021 via vaccinations and natural infections [40]. This has led to a continual decline in hospitalization rates in the US going back to March 2022 [41]. Long-term expansion of the PHE could result in a loss of public trust in other public health agencies. In addition, if the PHE is expanded indefinitely it could weaken the power of an emergency declaration and erode public trust for future crises.

## 7. Termination of Public and National Health Emergencies

Among those COVID-19 services that will be affected after the ending of PHE will include COVID-19 testing, vaccines, and treatments. For now, COVID-19 vaccines will remain free of charge from the federal government for all adults and children in the US. This may change based on government supplies of the vaccine. COVID-19 at-home tests may not be covered by insurance; however, the CDC will provide online access to a free test locating system to help the public find access to testing via the (ICATT) program. Paxlovid, an antiviral for COVID treatment, would be free of charge to the public until government supplies are exhausted. After supplies are exhausted treatments such as Paxlovid will no longer be free and the price of the medication will be determined by medication providers and by individual health insurance. On the date, ending the COVID-19 PHE declaration will transition the CDC’s authorizations to collect certain types of public health data [42]. Reporting frequency and certain data acquisitions will end or change. However, the CDC will continue to provide data required to ensure public safety, especially data to support at-risk populations in the US for the most severe complications of COVID-19 [42]. The CDC’s authorization to collect and receive certain types of data will change when the PHE ends. Among those metrics that the CDC will continue to provide, will include COVID-19 hospital admissions where all hospitals are required to report data through the end of April 2024; COVID-19 deaths, but the source of data will changed to include a new metric on the percent of deaths that COVID-19 deaths, but the source of data will changed to include additional analysis on the percent of deaths that are COVID-19-related along with vital statistics via the NVSS program. The weekly postings of ER patient visits will continue along with CDC regional COVID test positivity data as well as waste water and genomic surveillance [42]. The following data that will be removed by the CDC include COVID-19 case and mortality data will no longer be available via the COVID-19 Tracking system. Test positivity data will no longer be reported on the COVID-19 Electronic Reporting CELR system data will change when the PHE ends. Among those metrics that the CDC will continue to provide, will include COVID-19 hospital admissions where all hospitals are required to report data through the end of April 2024; COVID-19 deaths, but the source of data will changed to include a new metric on the percent of deaths that are COVID-19-associated, and other metrics from the National Vital Statistics System (NVSS) will be reported weekly; Emergency department patient visits with diagnosed COVID-19 will continue to be posted on a weekly basis; CDC reporting of COVID-19 test positivity via regional-level test positivity data from the National Respiratory and Enteric Virus Surveillance System (NREVSS); Wastewater surveillance and genomic surveillance; and Count of COVID-19 vaccines administered will remain for jurisdictions who continue to submit data updated monthly, instead of weekly [42]. The following data that will be removed by the CDC include COVID-19 case and death data will no longer be highlighted on COVID Data Tracker; National, county-level test positivity data from COVID-19 Electronic Reporting (CELR) will no longer be available; and the V-safe tracking system for health check-ins after vaccination health check-ins will end [42]. However, adverse events following vaccination will still be reported to the Vaccine Adverse Event Reporting System (VARES) [42].

## 8. Impact of termination on the COVID-19 related services among medically underserved populations in Tennessee

**Figure 3:**
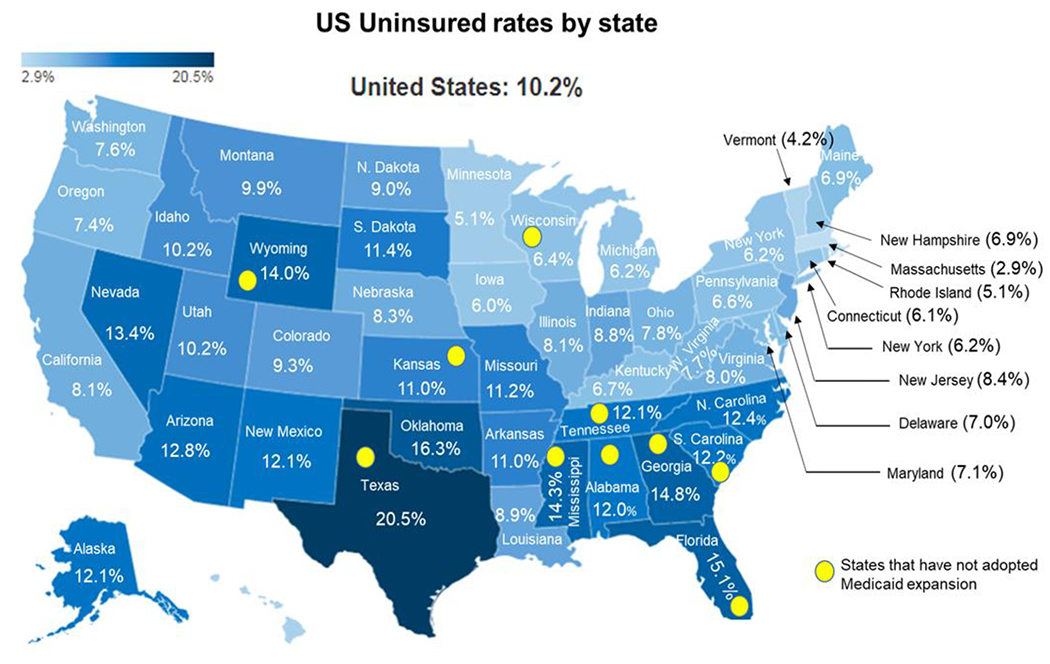
United States uninsured rates by state in 2022, and the status of state Medicaid expansion, Yellow circles represent states that have not adopted Medicaid expansion in 2023. Modified from the Kaiser Foundation map of US uninsured rates by state. Modified from Kaiser Foundation map of US uninsured rates by state in 2022 Status of State Medicaid Expansion Decisions: Interactive Map https://www.kff.org/medicaid/issue-brief/status-of-state-medicaid-expansion-decisions-interactive-map/ (accessed May 24, 2023).

**Figure 4:**
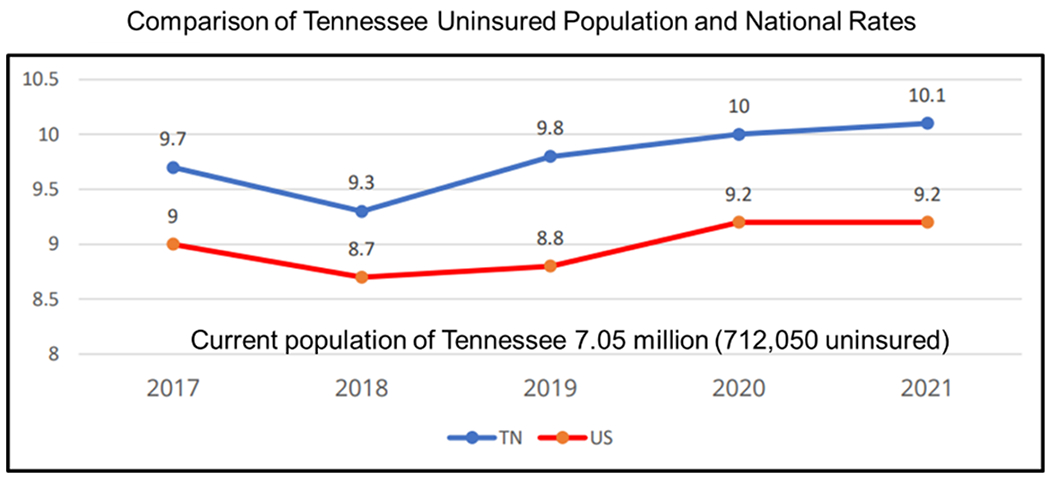
A comparison of Tennessee uninsured population and national rates from 2017 to 2021.

**Figure 5:**
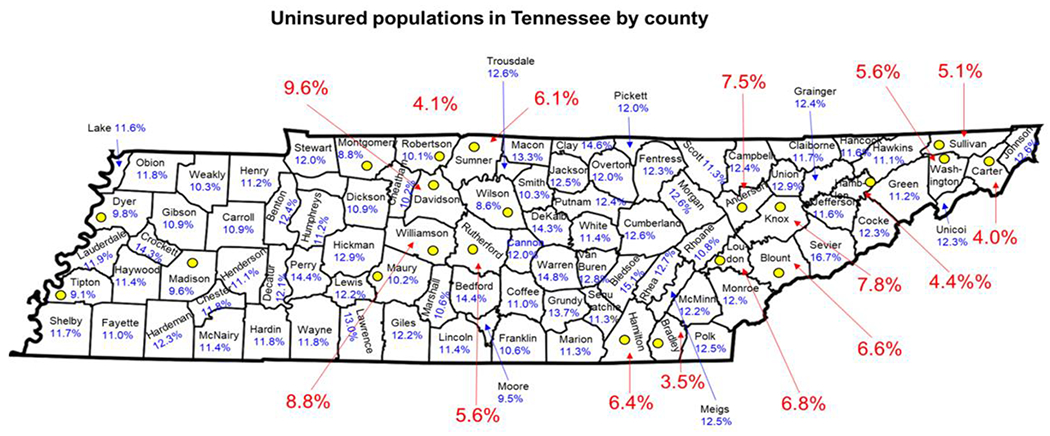
Uninsured populations in Middle Tennessee by county 2022. The yellow dots indicate counties with an uninsured rate of 10.2% or less (at or below the national average). Urban counties in Tennessee and their uninsured rates are shown in red.

After May 11, 2023, COVID vaccines and booster’s availability and access will depend on federally purchased vaccines. The vaccines and booster will remain free to everyone regardless of insurance coverage. Providers of these federally purchased vaccines will not be allowed to charge individuals based on coverage or network status [42]. As long as federal provided vaccines last, vaccine providers will provide vaccines free or charge to participants. Once federal supplied vaccines are exhausted, vaccines will continue to be provided free of charge to most individuals with public and private insurance [42]. The cost burden of vaccines will be placed on uninsured and underinsured populations in the US. This has been exacerbated by the reluctance of some states to adopt Medicaid expansion (Figure 3). States that have failed to expand Medicaid largely have higher uninsured rates (Figure 3). In addition, failed Medicaid expansion observed especially in the South has likely contributed to the higher rates of uninsured populations compared to other regions in the US. (Figure 3). In 2021, according to the America’s Health Rankings, published by the United Health Foundation revealed that 10.1% of Tennesseans were uninsured compared to the national rate of 9.2% [43]. With a current population of 7.05 million, 712,050 Tennesseans are without health insurance. This number does not include the number of underinsured people in Tennessee. Tennessee is ranked 37th among the 50 states for the percentage of the population that is uninsured [43]. Affordability is the most important reason why Tennesseans are unable to obtain health insurance [43]. The projected out-of-pocket expenses for COVID related services among uninsured and underinsured populations in Tennessee will likely lead to widespread financial hardships. This will exacerbate conditions on the ground during periods of heightened community spread and increased infection rates that will likely increase the number of hospitalizations and deaths over-time in Tennessee. The state of Tennessee uninsured rates have been consistently higher than the national average (Figure 4). The COVID-19 pandemic has temporarily reduced the number of uninsured populations in Tennessee due the emergency declarations. Ending of the PHE and NHE declarations could result over time in higher rates of uninsured populations in Tennessee. There are 95 counties in Tennessee and Middle Tennessee represents the largest and most populous region with a mix of both urban and rural counties. Those populations in Tennessee that are uninsured are more likely to live in rural counties when compared to urban counties (Figure 5). The majority urban/populous counties in Tennessee are below the national average 10.2% who are without medical insurance (Figure 5).

The waivers and flexibilities of the declarations were meant to expire and were not designed to be extended indefinitely. However, what we have learned in the past is that this pandemic continues to evolve and the SARS-CoV-2 can be unpredictable. The end of the emergency declarations also means the end of Title 42, a provision used by both the Trump and the Biden Administrations to expel migrants and asylum seekers coming from countries where there could be high burden COVID infections and low vaccination rates. Even more, ending the public health emergency declarations on May 11, 2023, data that has been crucial to understanding the spread and impact of COVID-19 will be reported by government sources and associated agencies less frequently, or is no longer reported at all.

**Figure 6:**
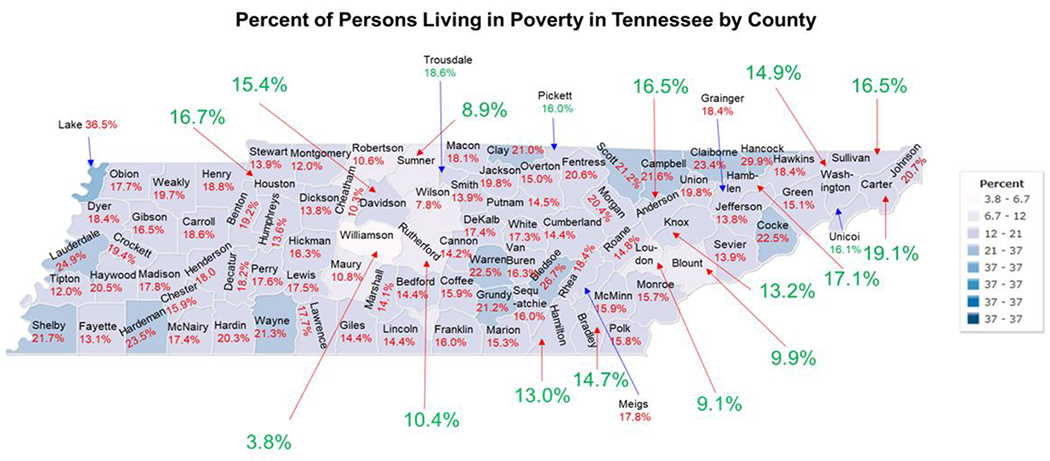
Percent of all persons living in poverty in Tennessee by county 2022. The yellow dots indicate the 17 urban counties in Tennessee and the percent poverty for urban counties is in blue text. The percent poverty in the 78 rural counties are shown in red text. A percent poverty table is also shown.

The state of Tennessee has a total of 95 counties including 17 urban counties and 78 counties designated as rural counties based on population and land development (Figure 6). A majority of the population of Tennessee resides in the 17 urban counties. The percentage of people living in poverty in urban counties in Tennessee ranges from 3.8% in Williamson County to 19.1% in Carter County (Figure 6) [44]. The percentage of people living in poverty in rural counties in Tennessee ranges from 7.8% in Wilson County to 36.5% in Lake County (Figure 6) [44]. Rural counties in Tennessee are more heavily impacted by poverty and have less access to COVID vaccines, and COVID services. Therefore, ending the PHE and NHE government services would more greatly impact the health status of residents of rural Tennessee (44). We have also previously reported higher COVID-19 vaccine hesitancy and lower COVID-19 vaccine uptake among populations residing in rural counties compared to urban counties in Tennessee [45,46].

## 9. Strategies to provide extended COVID services to medically underserved populations

The burden of having to pay for COVID related services after government supplies are exhausted and hospitalizations will largely impact those medically underserved populations that are elderly and are experiencing existing financial hardships. Strategies to provide extended COVID-19 services to medically underserved populations will likely include an extension of the previous declarations in some form. This will require legislative action by congress for a special allocation of funds that could be problematic with the currently divided congressional leadership that are highly opposed to additional government spending. To perform this task at scale the development of a community workforce employing community health care workers could be a viable alternative to primary care physicians for promoting and administering COVID-19 boosters, development and implementation COVID-19 awareness and education programs to reduce misinformation and vaccine hesitancy and improve vaccine confidence and uptake especially among those that are most vulnerable. The expansion of telehealth services including tele prescription services to include services provided regardless of location of the individual. We are aware that many communities in rural Tennessee may not have access to broadband service and would require telehealth access via mobile units that can be dispatched to those communities that lack transportation, phone, and internet service. This is especially important in rural communities where there is reduced healthcare infrastructure and a shortage of primary care physicians. In addition, extension of the Biden’s administration Health Equity Task that was developed to ensure an equitable COVID-19 pandemic response and recovery and preparedness for future pandemics. Achieving this goal will require long-term, sustained investments to address COVID health literacy, vaccine hesitancy, COVID care for the uninsured and those suffering from long COVID.

## 10. Limitations of the study

Limitations of this study include the poor documentation of ending of PHE and NHE declarations in the scientific literature and its impact on uninsured communities in Tennessee and the US. In addition, important details of the expired declarations have been limited to federal bulletins that are not readily accessible for review. The declarations were continually being extended and revised in accordance with changes in public health needs during the pandemic. The study is limited to the state of Tennessee however, the impact of ending the PHE and NHE declarations impacted many states across the US. Finally, the complexity of all the moving parts of establishing and ending these declarations were often poorly presented by government and state officials.

## 11. Conclusion

The National and Public Health emergency declarations were essential for greatly reducing morbidity and mortality associated with the COVID-19 pandemic in the US. These declarations created no cost COVID-19 service access for economically marginalized communities in the US that were most heavily impacted by the pandemic. Uninsured populations have increased, partly due to the failure to expand Medicaid in Tennessee, along with disproportionate poverty and vaccine hesitancy among rural communities has made Tennesseans more vulnerable to COVID-19 associated morbidity and mortality. It is essential that some form of the existing declarations must be maintained and or extended to ensure the health and wellness of medically underserved populations in Tennessee and the US.

## Data Availability

This manuscript did not report any laboratory-based data.

## References

[1] U.S. Department of Health and Human Services (HHS). Determination that a Public Health Emergency Exists. 2020. (accessed 10, January 2022]. https://www.phe.gov/emergency/news/healthactions/phe/Pages/2019-nCoV.aspx

[2] Fact Sheet: COVID-19 Public Health Emergency Transition Roadmap https://www.hhs.gov/about/news/2023/02/09/fact-sheet-covid-19-public-health-emergency-transitionroadmap.html#:~:text=Based%20on%20current%20COVID%2D19,day%20on%20May%2011%2C%202023. (accessed 9, February 2023).

[3] Sunshine G, Barrera N, Corcoran AJ, Emergency Declarations for Public Health Issues: Expanding Our Definition of Emergency. The Journal of law, medicine & ethics: a journal of the American Society of Law, Medicine & Ethics. 47 (2019): 95–99. 10.1177/1073110519857328 PMC6644713 31298138

[4] Cubanski Juliette, Kates Jennifer, Tolbert Jennifer, What Happens When COVID-19 Emergency Declarations End? Implications for Coverage, Costs, and Access Published: https://www.kff.org/coronavirus-covid-19/issue-brief/what-happens-when-covid-19-emergency-declarations-end-implications-for-coverage-costs-and-access/# published (accessed 31, January 2023).

[5] Kaiser Family Foundation: Many Uninsured People Could Lose Access to Free COVID-19 Testing, Treatment, and Vaccines as Federal Funding Runs Out https://www.kff.org/uninsured/press-release/many-uninsured-people-could-lose-access-to-free-covid-19-testing-treatment-and-vaccines-as-federal-funding-runs-out/ (Accessed 28 March 2022)

[6] 2021 America’s Health Rankings, United Health Foundation. https://www.americashealthrankings.org/search?q=US+uninsured+rates

[7] The Kaiser Family Foundation, State Health Care Snapshots: Tennessee. Available online: https://www.kff.org/statedata/election-state-fact-sheets/tennessee/ (accessed on 15 October 2020).

[8] Center for Disease Control and Prevention, National Center for Health Statistics. Quarterly Provisional Death Rates for COVID-19. Available online: https://www.cdc.gov/nchs/pressroom/sosmap/covid19_mortality/Provisional_COVD19.htm (accessed on 7 March 2023).

[9] Death Rates from COVID-19 in the United States as of March 10, 2023, by State (per 100,000 People). Available online: https://www.statista.com/statistics/1109011/coronavirus-covid19-death-rates-us-by-state/ (accessed on 10 March 2023).

[10] The Tennessean: Why Does Tennessee Have So Many Uninsured People? By Refusing to Expand Medicaid, the State’s Political Leadership Has Made a Decision to Let Lower-Income White, Black and Latino Residents Suffer. Available online: https://www.tennessean.com/story/opinion/2022/04/06/why-does-tennessee-have-so-many-uninsured-people/9484161002/ (accessed on 6 April 2022).

[11] Cosby AG, McDoom-Echebiri MM, James W, Growth and Persistence of Place-Based Mortality in the United States: The Rural Mortality Penalty. Am. J. Public Health 109 (2019): 155–162. 30496008 10.2105/AJPH.2018.304787 PMC6301407

[12] Singh GK, Siahpush M Widening rural-urban disparities in life expectancy, U.S.; 1969–2009. Am. J. Prev. Med 46 (2014): e19–e29. 10.1016/j.amepre.2013.10.017 24439358

[13] Conway BN, Han X, Munro HM, The obesity epidemic and rising diabetes incidence in a low-income racially diverse southern US cohort. PLoS ONE 13 (2018): e0190993. 29324894 10.1371/journal.pone.0190993 PMC5764338

[14] Artiga S; Damico A Health and Health Coverage in the South: A Data Update. 10 February 2016. Available online: https://www.kff.org/racial-equity-and-health-policy/issue-brief/health-and-health-coverage-in-the-south-a-data-update/#:~:text=Southerners%20are%20more%20likely%20to,the%20rest%20of%20the%20country (accessed on 10 February 2016).

[15] Kaiser Family Foundation: Jennifer Kates Juliette Cubanski, Cynthia Cox and Jennifer Tolbert. Timeline of End Dates for Key Health-Related Flexibilities Provided Through COVID-19 Emergency Declarations, Legislation, and Administrative ActionsTimeline of End Dates for Key Health-Related Flexibilities Provided Through COVID-19 Emergency Declarations, Legislation, and Administrative Actions. https://www.kff.org/coronavirus-covid-19/issue-brief/timeline-of-end-dates-for-key-health-related-flexibilities-provided-through-covid-19-emergency-declarations-legislation-and-administrative-actions/ (accessed 28, April 2023)

[16] National and World News: Public health vs. national emergencies: what are they, when have they happened? https://www.pennlive.com/nation-world/2017/10/public_health_and_national_eme.html (accessed on 26, October 2017).

[17] Medicaid and CHIP Payment and Access Commission: Federal emergency authorities. https://www.macpac.gov/subtopic/federal-emergency-authorities/

[18] FEMA: Eligible Emergency Protective Measures. https://www.fema.gov/fact-sheet/eligible-emergency-protective-measures (accessed on 10 May 2023).

[19] Health Insurance Portability and Accountability Act of 1996. Office of Assistant Secretary for Planning and Evaluation (ASPE) https://aspe.hhs.gov/reports/health-insurance-portability-accountability-act-1996 (accessed 20 August 1996).

[20] Holshue ML, DeBolt C, Lindquist S, Washington State 2019-nCoV Case Investigation Team. First Case of 2019 Novel Coronavirus in the United States. N. Engl. J. Med 10 (2020): 929–936. 10.1056/NEJMoa2001191 PMC7092802 32004427

[21] National Archives: The Federal Register: Regulatory Relief To Support Economic Recovery; Request for Information (RFI), Health and Human Services Department. https://www.federalregister.gov/documents/2020/11/25/2020-25812/regulatory-relief-to-support-economic-recovery-request-for-information-rfi (accessed on 25 November 2020.

[22] HHS. Determination that a public health emergency exists. 2020a. https://www.phe.gov/emergency/news/healthactions/phe/Pages/2019-nCoV.aspx. (accessed on 01 September 2021).

[23] CMS.gov Centers for Medicare & Medicaid Services: Current Emergencies https://www.cms.gov/about-cms/agency-information/emergency/epro/current-emergencies/current-emergencies-page (accessed on April 26, 2023).

[24] CRS. Emergency authorities under the National Emergencies Act, Stafford Act, and Public Health Service Act. 2020c. [October 15, 2021]. https://crsreports.congress.gov/product/pdf/R/R46379.

[25] Zhu N, Zhang D, Wang W, , China Novel Coronavirus Investigating and Research Team 2020. A novel coronavirus from patients with pneumonia in China. N. Engl. J. Med 382 (2019): 727–733. 31978945 10.1056/NEJMoa2001017 PMC7092803

[26] Dhama K, Khan S, Tiwari R, Coronavirus disease 2019–COVID-19. Clin. Microbiol. Rev 33 (2020), e00028–20. 32580969 10.1128/CMR.00028-20 PMC7405836

[27] Guan WJ, Ni ZY, Hu Y, , China Medical Treatment Expert Group for Covid-19 (2020). Clinical Characteristics of Coronavirus Disease 2019 in China. N. Engl. J. Med 18 (2020): 1708–1720. 10.1056/NEJMoa2002032 PMC7092819 32109013

[28] Fu L, Wang B, Yuan T, Clinical characteristics of coronavirus disease 2019 (COVID-19) in China: A systematic review and meta-analysis. The Journal of infection 6 (2020): 656–665. 10.1016/j.jinf.2020.03.041 PMC7151416 32283155

[29] Stein SR, Ramelli SC, Grazioli A, SARS-CoV-2 infection and persistence in the human body and brain at autopsy. Nature 7941 (2022): 758–763. 10.1038/s41586-022-05542-y PMC9749650 36517603

[30] Zhou F, Yu T, Du R, Clinical course and risk factors for mortality of adult inpatients with COVID-19 in Wuhan, China: a retrospective cohort study. Lancet (London, England) 10229 (2020): 1054–1062. 10.1016/S0140-6736(20)30566-3 PMC7270627 32171076

[31] Martínez-Córdoba PJ, Benito B, García-Sánchez IM Efficiency in the governance of the Covid-19 pandemic: political and territorial factors. Globalization and health 1 (2021): 113. 10.1186/s12992-021-00759-4 PMC8454294 34548073

[32] Coronavirus disease (covid-2019) situation reports [internet]. Available from: [https://www.who.int/emergencies/diseases/novel-coronavirus-2019/situation-reports/]

[33] Centers for Medicare & Medicaid Services (CMS.gov): Trump Administration Makes Sweeping Regulatory Changes to Help U.S. Healthcare System Address COVID-19 Patient Surge. https://www.cms.gov/newsroom/press-releases/trump-administration-makes-sweeping-regulatory-changes-help-us-healthcare-system-address-covid-19 (accessed on 30 March 2020).

[34] Medicaid.GOV: Section 1135 Waiver Flexibilities: CMS 1135 Waivers https://www.medicaid.gov/resources-for-states/disaster-response-toolkit/section-1135-waiverflexibilities/index.html#:~:text=Under%20section%201135%20of%20the,by%20a%20federally%2Ddeclared%20PHE. (accessed on 10 August 2023).

[35] New York Times: Where $5 Trillion in Pandemic Stimulus Money Went Alicia Parlapiano, Deborah B. Solomon, Madeleine Ngo and Stacy Cowley https://www.nytimes.com/interactive/2022/03/11/us/how-covid-stimulus-money-was-spent.html (accessed on 11 March 2022)

[36] Chalkias S, Harper C, Vrbicky K, A Bivalent Omicron-Containing Booster Vaccine against Covid-19. N. Engl. J. Med 14 (2022): 1279–1291. 10.1056/NEJMoa2208343 PMC9511634 36112399

[37] Najjar-Debbiny R, Gronich N, Weber G, Effectiveness of Paxlovid in Reducing Severe Coronavirus Disease 2019 and Mortality in High-Risk Patients. Clinical infectious diseases: an official publication of the Infectious Diseases Society of America 3 (2023): e342–e349. 10.1093/cid/ciac443 PMC9214014 35653428

[38] Silk BJ, Scobie HM, Duck WM, COVID-19 Surveillance After Expiration of the Public Health Emergency Declaration - United States, May 11, 2023. MMWR Morb. Mortal Wkly. Rep 19 (2023): 523–528. 10.15585/mmwr.mm7219e1 PMC10208372 37167154

[39] Ahmad FB, Cisewski JA, Xu J, Provisional Mortality Data - United States, 2022. MMWR. Morbidity and mortality weekly report 18 (2023): 488–492. 10.15585/mmwr.mm7218a3 PMC10168603 37141156

[40] Jones JM, Opsomer JD, Stone M, Updated US infection- and vaccine-induced SARS-CoV-2 seroprevalence estimates based on blood donations, JAMA 328 (2022), 298–301. 35696249 10.1001/jama.2022.9745 PMC9194752

[41] CDC. COVID-19 data review: update on COVID-19-related mortality. Atlanta, GA: US Department of Health and Human Services, CDC; 2023. Accessed April 14, 2023. https://www.cdc.gov/coronavirus/2019-ncov/science/data-review/index.html

[42] CDC. End of the Federal COVID-19 Public Health Emergency (PHE) Declaration. https://www.cdc.gov/coronavirus/2019-ncov/your-health/end-of-phe.html#:~:text=May%2011%2C%202023%2C%20marks%20the,to%20the%20COVID%2D19%20pandemic. (accessed on 5 May 2023)

[43] America’s Health Ranking: United Health Foundation. Uninsured in Tennessee. https://www.americashealthrankings.org/explore/measures/HealthInsurance/TN (accessed on 10 August 2023.

[44] Index Mundi Person in Poverty in Tennessee. https://www.indexmundi.com/facts/united-states/quick-facts/tennessee/population#map access on July 1, 2022.

[45] Alcendor DJ, Matthews-Juarez P, Williams N, COVID-19 Vaccine Hesitancy and Uptake among Minority Populations in Tennessee. Vaccines, 11 (2023): 1073. 37376464 10.3390/vaccines11061073 PMC10302928

[46] Alcendor DJ Targeting COVID Vaccine Hesitancy in Rural Communities in Tennessee: Implications for Extending the 540 COVID-19 Pandemic in the South. Vaccines, 9 (2021): 1279. 34835210 10.3390/vaccines9111279 PMC8621887

